# Level of awareness and treatment of anxiety and depression during pregnancy in southeast Nigeria

**DOI:** 10.4102/sajpsychiatry.v24i0.1192

**Published:** 2018-11-08

**Authors:** Ezeme M. Sunday, Paul C. Okoli, Vincent O. Dinwoke

**Affiliations:** 1 Department of Psychiatry, Enugu State University Teaching Hospital, Nigeria; 2 Department of Obstetrics and Gynaecology, Enugu State University Teaching Hospital, Nigeria

## Abstract

**Background:**

Anxiety and depressive disorders are somewhat masked by features of pregnancy; hence many women are ignorant of them and are untreated.

**Aim:**

To determine the level of awareness and treatment of anxiety and depression in pregnancy.

**Setting:**

The study was carried out at the antenatal clinic of Enugu State University Teaching Hospital, Enugu, Nigeria.

**Method:**

This was a cross-sectional and descriptive study of 200 pregnant women in consecutive attendance of the antenatal clinic using the Hospital Anxiety and Depression Scale (HADS) and a sociodemographic questionnaire.

**Results:**

Of the participants, 23.5% had anxiety and/or depression, 7.5% of them were aware of their condition and only 0.5% of all the participants or 6.7% of those who were aware of their problem received treatment.

**Conclusion:**

Anxiety and depression are prevalent among pregnant women. Because of overlap of symptoms of anxiety and depression with those of pregnancy, the awareness is very low; hence many of them suffer immensely without treatment.

## Introduction

One of the crucial milestones in a woman’s life is pregnancy and childbirth, especially in Africa. Most women look forward to becoming mothers and have positive expectations while pregnant. However, because of the physical, hormonal, neurotransmitter and psychosocial changes that occur, pregnancy can be stressful and overwhelming, and many women present with signs and symptoms. Pregnant women can experience fatigue, sluggishness, heartburn and indigestion, nausea and vomiting (morning sickness), backache, headache, breathlessness, tingling and numbness in the hands, frequent urination, moodiness and crying spells,^1^ dizziness and fainting, cravings and aversion to certain foods. Some of them may be unduly worried, fearful and preoccupied with thoughts about whether they will be a good mother; whether the child may be malformed;^3^ whether there may be exacerbation of pre-existing medical or psychological illnesses; how they will cope with consequent financial challenges; and feelings of being less attractive.^4^

Similarly, persons with anxiety disorder may present with features like worries about impending doom, fearfulness, poor attention, forgetfulness, headache, abdominal discomfort, indigestion, fatigue, sweating, tremulousness, breathlessness, tingling and numbness, fainting and dizziness in absence of any other psychiatric or organic disorder. Moreover, individuals with depressive disorder may be experiencing persistent low mood, crying spells, body weakness, poor appetite, loss of interest in previously enjoyed activities, feelings of worthlessness, poor attention, poor memory, loss of sexual interest, and suicidal thoughts or acts.^5^

Pregnancy and anxiety and or depression affect each other and share certain attributes in common:^6^

- Women who are already living with chronic stress may find themselves unable to cope with the additional demands of pregnancy.
- Women who are living in poverty and/or who already have many children may perceive pregnancy with ambivalence and negative feelings.
- In relationships that are under pressure, domestic violence tends to increase during pregnancy and raises the chance of developing anxiety and depression.
- Pregnancy-related sex steroids increase the activation of the hypothalamic–pituitary–adrenal axis (cortisol stress system), which is associated with anxiety and depression. Some researchers have opined that elevated levels of cortisol may affect foetal growth and development and may be associated with altered temperament and behavior.^7^

It is worthy of note that anxiety and depressive disorders commonly coexist and the symptoms occur at most times of the day; they are distressful and impair work and interpersonal functioning. Hence they most times require the attention of professional mental health workers, unlike the similar features found in pregnancy, which usually vary with environmental stimuli and are relieved by mere social support and reassurance. The signs and symptoms of antenatal anxiety and depression are not different from those of non-pregnant women. However, psychological symptoms like irrational worries and fear, anhedonia, self-blame, hopelessness and worthlessness seem to be more reliable in pregnancy than the somatic features, such as sweating, breathlessness, fatigue, body ache, headache, dizziness and fainting, which may impair detection and subsequent treatment of the disorders.

Antenatal anxiety and depression are a major public health problem because of their high rate of occurrence.^8,9,10^ A WHO^10^ estimate showed that depression affected 3.9%, that is, 7 million Nigerians.^11^ In a computer assisted survey of the public’s views of mental health in pregnant and post-partum women, using a random sampling of 1207 participants from the province of Alberta, Canada, 70.5% had knowledge of prenatal mental health and only 26.6% of the participants accurately identified that anxiety and depression in pregnancy negatively impacts on child development.^12^ Using the Center for Epidemiological Studies – Depression (CES-D) to screen 3472 pregnant women in a cross-sectional study, it was found that 20% were depressed while only 13.8% reported receiving any formal treatment for depression.^13^ Even though there is evidence-based treatment, patients with depression do not usually get the help they need. Despite high disease burden, perinatal depression remains undertreated in most African countries, including Nigeria.^14,15^ In a Nigerian study of 2152 participants aged 65 and above in a face-to-face interview using the WHO Composite International Diagnostic Interview, only 37% of the elderly patients received any form of treatment for depression.^16^ However, when prenatal mental health problems are untreated, over one-third of pregnant women may experience unremitting symptoms that persist into the post-partum period (the child’s preschool years).^17^ Despite a series of consultations with healthcare officers during pregnancy, the vast majority of women do not seek help for symptoms of anxiety, depression and stress or voluntarily disclose their symptoms.^18,19^ Some women have reported not obtaining timely referral^20^ as a reason for not seeking help, while others have cited deeply held views about psychiatric illness in the prenatal and postnatal periods, like the expectation to be constantly happy because one is pregnant and/or about to have a baby; the attribution of mental disorders as maternal incompetency and being a failure as a mother; the fear that others will think less of them after admitting to having features of anxiety and depression; the desire to be a ‘super mother’; and the belief that the illness will resolve spontaneously.^21,22,23^ In general, individuals with mental disorders usually experience feelings of discrimination, stigma and shame. In addition, most Nigerians lack awareness and knowledge about mental health and available mental health services. With the grossly inadequate mental health services, especially in rural areas, financial handicaps become an additional barrier to obtaining care.^24^

The impact of anxiety and depression in a pregnant woman can be enormous and often predispose women to postnatal depression,^25^ and maternal depressive symptomatology has been shown to affect the mother’s responsiveness to the child,^26^ adequate immunisation, breastfeeding and emotional support. Other members of the family may also be affected, concerning good parenting, interpersonal relationships and finances. Maternal mood symptoms during pregnancy have been linked to incidences of low birth weight, premature delivery,^27,28^ behavioural problems, and delayed cognitive and linguistic development of the child.^29,30,31^ A large number of preterm births are not predicted or explained by known obstetric risk factors.^32^ Approximately more than one-third of adverse birth outcomes are not predicted by prenatal obstetric risk assessments.^33^ As a result, scholars have considered other circumstances in women’s lives that may be responsible for shortened gestation. Currently, a body of systematic research has reported convincing evidence that maternal stress and anxiety contribute to some cases of preterm birth.^34,35^ Even when a child is not delivered preterm, gestational anxiety tends to exhibit lasting adverse effects on the behavioural and psychological well-being of the foetus. The infants of women with high pregnancy anxiety show worse cognitive and motor performance,^36^ as well as poor attention regulation, when compared with those of mothers with low gestation anxiety scores.^37^ Furthermore, in a cohort study of pregnant women, high anxiety scores predicted negative temperament in their children of 2 years old, poorer executive function and decreased grey matter density in areas associated with cognitive function at 6–9 years of age.^38^

With the remarkable broad overlap and similarities in clinical presentation of anxiety and depression and the signs and symptoms of pregnancy, many pregnant women and their health workers are unaware of their problems and hence they remain untreated. This study aimed at highlighting the level of awareness and treatment of anxiety and depression among pregnant women.

## Methods

### Study design

This was a cross-sectional descriptive study of consecutive attendees of pregnant women who came for routine antenatal care.

### Study setting

The study was carried out at the antenatal clinic of the Department of Obstetrics and Gynaecology, Enugu State University Teaching Hospital, Enugu. The clinic is mainly attended by women within the Enugu metropolis and by a few referrals from the rural areas.

### Study population and sampling strategy

Consecutive attendees of pregnant women who came for a routine check-up at the antenatal clinic were used for the study. Informed consent to take part in the study was obtained before questionnaires were administered to each participant irrespective of gestational age, parity or obstetrics and gynaecological history. In collaboration with other hospital staff, 200 participants were recruited for the study.

### Data collection

A sociodemographic questionnaire and the Hospital Anxiety and Depression Scale (HADS) were used to collect data. The sociodemographic questionnaire gave information about the participant’s age, employment status, educational attainment, parity, gestational age, obstetric history and knowledge and treatment of anxiety and depression.

The HADS is a 14-item scale, seven of which relate to anxiety and the other seven determine depression. Each item of the questionnaire is scored from 0 to 3. Hence an individual may have a total score between 0 and 21 for either anxiety or depression. The cut-off point has been taken to be 8/21 for either anxiety or depression. For anxiety, this gave a specificity and sensitivity of 0.78 and 0.9, respectively, while for depression it gave a specificity and sensitivity of 0.79 and 0.83, respectively. A score of 8–10 is considered a borderline case, while a score of 11–21 is regarded as an abnormal case. This instrument has been validated and used in Nigeria.^39^

### Data analysis

Data obtained were analysed using SPSS version 16. The frequency distribution of the variables was calculated.

## Ethical consideration

Approval to conduct this study was obtained from the Ethical Committee of Enugu State University Teaching Hospital before data collection commenced.

## Results

### Study population

A total of 200 participants attending the antenatal clinic were studied.

### Sociodemographic characteristics

The age of the participants ranged from 17 to 41 years, with a mean of 29.3 ± 4.4 years. One hundred and ninety-nine (99.5%) of them were married and 1 (0.5%) of them was unmarried. Almost half of them were employed and their minimum educational attainment was secondary school education.

**TABLE 1:** Sociodemographic variables of the participants.

Variable		Frequency	%
Marriage	Married	199	99.5
	Unmarried	1	0.5
Employment	Employed	101	50.5
	Unemployed	99	49.5
Highest level of education	Secondary	23	11.5
	Tertiary	177	88.5
Age range (years)	24	-	-
Minimum age (years)	17	-	-
Maximum age (years)	41	-	-
Mean age (years)	29.3	-	-
Standard deviation	±4.4	-	-

**TABLE 2:** Awareness and treatment of anxiety and/or depression among the participants.

Variable	Frequency	%
Neither anxious nor depressed	137	68.5
Anxious and/or depressed	47	23.5
Awareness of anxiety and/or depression	15	7.5
Receiving treatment (antidepressant)	1	0.5

### Anxious and/or depressed

Forty-seven (23.5%) participants were found to be anxious and/or depressed, while 137 (68.5%) were neither anxious nor depressed.

### Awareness of anxiety and depression

Fifteen (7.5%) participants were aware of their psychological problem.

### Treatment of anxiety and depression

Only 1(0.5%) of all the participants or 6.7% of those aware of their problem were receiving treatment with antidepressants.

## Discussion

In a cross-sectional survey of 314 pregnant women attending antenatal clinics at Abeokuta North Local Government, Nigeria, using the Edinburgh Postnatal Depression Scale, a prevalence for antenatal depression of 24.5% was found.^40^ The result of this study showed that approximately one-quarter of the pregnant women were anxious and/or depressed. This is comparable to a prevalence of 18.0% of pregnant women found with anxiety and/or depression in a community study at Hyderabad, Pakistan, involving 1368 pregnant women.^41^ However, a hospital-based cross-sectional study of 165 pregnant women using the HADS in Karachi, Pakistan, reported that 70.0% of them had anxiety and/or depression.^42^ The lower rate of anxiety and/or depression in this study might partly be because of some sociocultural differences existing within the different study populations.

About one-quarter of the participants were anxious and/or depressed, less than one-tenth knew about their psychological health status and only 0.5% of them were receiving treatment. This is far lower than what was reported in Alberta, Canada – that 70.5% of pregnant women had knowledge of prenatal mental health and 26.6% of them were able to identify the negative impact of anxiety and/or depression on the foetus.^12^ These findings confirm the finding of the research, which reported that antenatal depression is neither recognised nor adequately treated in our environment.^43^ In view of the fact that the signs and symptoms of anxiety and/or depression and pregnancy mimic one another, it is not surprising that the majority of women not understand their problems and would continue to endure them as a burden of pregnancy. The lack of requisite clinical skills to detect features of anxiety and depression by health workers in the antenatal clinics may also have contributed immensely to the lack of recognition of the symptoms and signs of anxiety and depression, especially in subsyndromal cases.^44,45^ Reluctance to disclose symptoms by patients, stigma, discrimination and the negative feelings people have towards individuals with mental illness may also have contributed to the poor awareness and treatment of anxiety and/or depression in pregnancy.

Some studies using a variety of depression instruments reported antenatal depression prevalences of 9.0% – 28.0% for predominantly middle-class women^46,47^ and 25% – 50%^41,42^ for low-income populations. In other words, the rate of occurrence of depression may be far higher in the rural areas that constitute the greater proportion of our population than in the urban area where this study was conducted. Consequentially, one may also expect the level of awareness of depression and anxiety among pregnant women and health workers in the rural areas to be much lower than found in the urban setting.

Based on the results of this research, it is glaringly obvious that measures should be put in place to enhance the education, detection and treatment of anxiety and depression in pregnant women. Pregnant women should be educated about the signs and symptoms of anxiety and depression while health workers at antenatal clinics should be involved in both education and training programmes to facilitate detection and treatment of anxiety and depression in pregnancy.

## Limitations of the study

This study was a cross-sectional study in which the participants were seen only once. The differences in age of the participants, gestational age and parity were not taken into consideration. The HADS is a self-administered questionnaire, and the information about awareness and treatment of anxiety and/or depression was self-reported. These factors may in one way or the other have affected the results obtained in this study.

## Conclusion

Anxiety and depressive disorders are prevalent among pregnant women, with associated distress and adverse foetal outcomes. It is difficult for patients and health workers to distinguish them amidst the signs and symptoms of pregnancy. Hence, all hands should be on deck to improve awareness and treatment by incorporating screening for anxiety and depression into routine antenatal care, especially in developing countries like Nigeria.
